# Serologic Markers for Ebolavirus Among Healthcare Workers in the Democratic Republic of the Congo

**DOI:** 10.1093/infdis/jiy499

**Published:** 2018-09-19

**Authors:** Nicole A Hoff, Patrick Mukadi, Reena H Doshi, Matthew S Bramble, Kai Lu, Adva Gadoth, Cyrus Sinai, D’Andre Spencer, Bradley P Nicholson, Russell Williams, Matthias Mossoko, Benoit Ilunga-Kebela, Joseph Wasiswa, Emile Okitolonda-Wemakoy, Vivian H Alfonso, Imke Steffen, Jean-Jacques Muyembe-Tamfum, Graham Simmons, Anne W Rimoin

**Affiliations:** 1 Department of Epidemiology, Fielding School of Public Health, University of California, Los Angeles; 2 Institut National de Recherche Biomédicale, Washington, District of Columbia; 3 Faculté de Médecine, Université de Kinshasa, Democratic Republic of the Congo (DRC), Washington, District of Columbia; 4 Department of Genetic Medicine Research, Children’s Research Institute, Children’s National Medical Center, Washington, District of Columbia; 5 Blood Systems Research Institute, San Francisco; 6 Department of Laboratory Medicine, University of California, San Francisco; 7 Molecular Epidemiology Research Laboratory, Veterans Affairs Medical Center, Durham, North Carolina; 8 University of California, Los Angeles-DRC Research Program; 9 Direction de lutte contre la Maladie, Ministère de la Santé Publique; 10 Ecole de Sante Publique, Université de Kinshasa, DRC

**Keywords:** Ebola virus, serology, Democratic Republic of the Congo, healthcare workers

## Abstract

Healthcare settings have played a major role in propagation of Ebola virus (EBOV) outbreaks. Healthcare workers (HCWs) have elevated risk of contact with EBOV-infected patients, particularly if safety precautions are not rigorously practiced. We conducted a serosurvey to determine seroprevalence against multiple EBOV antigens among HCWs of Boende Health Zone, Democratic Republic of the Congo, the site of a 2014 EBOV outbreak. Interviews and specimens were collected from 565 consenting HCWs. Overall, 234 (41.4%) of enrolled HCWs were reactive to at least 1 EBOV protein: 159 (28.1%) were seroreactive for anti-glycoprotein immunoglobulin G (IgG), 89 (15.8%) were seroreactive for anti-nucleoprotein IgG, and 54 (9.5%) were VP40 positive. Additionally, sera from 16 (2.8%) HCWs demonstrated neutralization capacity. These data demonstrate that a significant proportion of HCWs have the ability to neutralize virus, despite never having developed Ebola virus disease symptoms, highlighting an important and poorly documented aspect of EBOV infection and progression.

## Teaser

Healthcare workers in an area with a previous Ebola outbreak in the Democratic Republic of the Congo exhibited both antibody response and neutralizing capacity to at least 1 Ebola protein, despite never having been diagnosed with Ebola virus disease.

**(See the Editorial Commentary by Garry on pages 511–3.)**

Outbreaks of Ebola virus disease (EVD) represent a major public health challenge in sub-Saharan Africa. EVD was initially recognized in 1976 during simultaneous outbreaks in Zaïre (now Democratic Republic of the Congo [DRC]) (*Zaïre ebolavirus* [EBOV]) and Sudan (*Sudan ebolavirus*) [1, 2]. Since then, there have been >37 EVD outbreaks spanning 14 different countries in Africa, Europe, Asia, and North America, with the majority caused by EBOV, 8 of which (including an outbreak that is currently unfolding at the time of manuscript submission, May 2018) have occurred in the DRC [3]. During these outbreaks, there was increased infection and deaths among those healthcare workers (HCWs) charged with the responsibility of caring for those suffering with EVD, a contributing factor to outbreak progression [4].

Healthcare workers are on the front line of patient care, and thus at increased risk of disease acquisition due to occupational exposures to bodily fluids, lack of infection control training, and a dearth of personal protective equipment. Human-to-human transmission of ebolavirus occurs through direct contact with bodily fluids (vomit, diarrhea), secretions, or blood of infected people via broken skin or mucous membranes; it may also be transmitted through contact with contaminated surfaces and materials (eg, bedding, clothing) [5–9]. This risk is even greater in limited resource settings of sub-Saharan Africa and has been documented in several studies assessing the seroprevalence of blood-borne pathogens in these populations [10–13]. Historically, HCWs have perpetuated the spread and amplification of EVD and serve as axes of viral transmission, often before ebolavirus is even recognized as the causative agent [14–21]. The symptoms of EVD are frequently nonspecific, characterized by fever, headache, fatigue, muscle pain, vomiting, diarrhea, and abdominal pain [22], and can easily be confused with other endemic diseases such as typhoid or malaria [23]. The difficulties associated with clinical recognition and diagnostic capabilities make prevention efforts for HCWs complex because infected workers may transmit disease before any symptoms are accurately diagnosed.

Despite HCWs’ increased risk of acquiring and transmitting the disease, there is limited research assessing the total burden of ebolavirus among HCWs [10, 15–18, 24]. Understanding serologic responses in high-risk populations, such as HCWs, and determining overall seroprevalence in areas with previous EVD outbreaks may provide more information on exposure. Current literature on HCWs and serological testing for ebolaviruses (for evidence of exposure) is restricted mainly to anti-glycoprotein (GP) antibody, the viral protein critical for attachment to and penetration of host cells. Because of its positioning on the virion surface, GP is a target of neutralizing antibodies and has been frequently studied as a target for vaccines and other therapeutics [22, 25–27]. Furthermore, Richardson et al suggest limiting the definition of EBOV seropositivity to anti-GP reactivity based on a comparison of anti-GP and anti-nucleoprotein (NP) antibody responses [28]. However, a separate experiment by Becquart and colleagues in 2014, comparing sera from asymptomatic seropositive individuals to symptomatic survivors of EVD, showed that immunoglobulin G (IgG) responses were qualitatively different in each group: The asymptomatic group displayed a larger response to EBOV matrix protein (VP40), whereas the survivors had greater IgG responses to GP [29]. These findings indicate that anti-GP alone may not be sufficient as a marker for demonstration of previous exposure, especially in asymptomatically infected or otherwise unrecognized EVD survivors.

Here, we report the results of a serological survey of a sample of HCWs living in or near Boende, DRC, the site of an EVD outbreak that occurred 1 year prior to sample collection, in which at least 8 of the 68 (14%) reported cases were HCWs. We paired epidemiologic data with serologic detection of EBOV GP, anti-NP, and VP40, as well as microneutralization using EBOV pseudovirions to assess the presence of a range of anti-ebolavirus antibodies in this high-risk population.

## METHODS

### Enrollment

**Figure 1.**
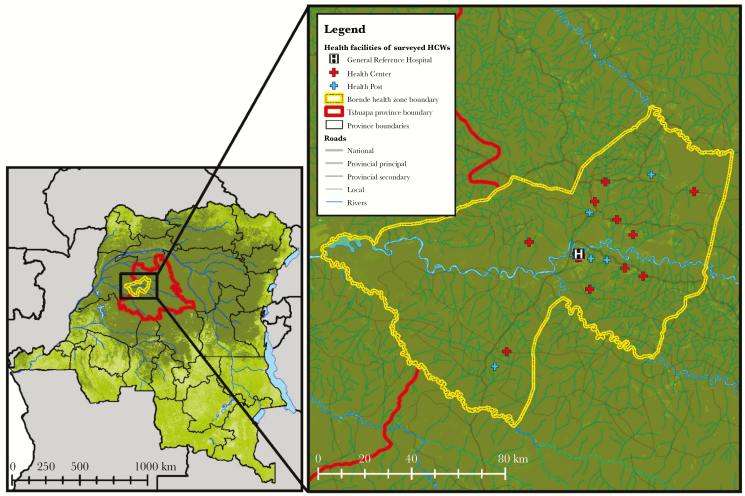
Health facility locations of surveyed healthcare workers in Boende health zone, Tshuapa province, Democratic Republic of the Congo. Abbreviation: HCW, healthcare worker.

From September to November 2015, we conducted a serosurvey in the Boende health zone, located 1200 km to the northwest of Kinshasa (capital of DRC) in the province of Tshuapa (Figure 1). All individuals who met the eligibility criteria (>18 years old, healthy [no fever or other illness reported at time of enrollment], and working in a health facility, and reported being actively involved during the 2014 Boende outbreak response) were approached for enrollment in the study. All participants were screened for signs of current illness or fever; no participants included in the current analysis reported ever having been or suspected of having been infected with EBOV. In total, 565 HCWs were consented and enrolled in the study from 26 health facilities (hospitals, health centers, or health posts), and 1 church, which provided health services to the community. The study included a sociodemographic and epidemiologic questionnaire and sample collection. Blood specimens frozen, stored and tested were obtained from all consenting participants by venipuncture into red-top Vacutainer tubes (BD Biosciences). After processing, aliquots of serum were frozen and stored at the Institut National de Recherche Biomedicale in Kinshasa and shipped to the Blood Systems Research Institute in San Francisco, California, for performance of the remaining serological and neutralization assays.

### Healthcare Worker Classification

Classifications were based on the World Health Organization system of classification [14, 30]. “Formal HCWs” were defined as individuals who work in health services. This includes clinical staff (physicians, nurses, and laboratory staff) and administrative workers in a hospital or health center, including cleaners, drivers, security, and community health workers. “Informal HCWs” were defined as traditional healers, pastors, and other nontraditional individuals providing care. HCWs were classified by their potential exposure to patients based on their reported occupation. “Direct contact” was defined as any HCW who had direct contact with sick patients and included doctors, nurses, and traditional healers. “Indirect contact” was defined as any HCW who did not have direct contact with sick patients, but had contact with biologic specimens, patient materials, or family members of sick patients (eg, laboratory technicians and room cleaners). “Unlikely contact” was defined as any HCW who was unlikely to have contact with sick patients and included positions such as hospital guards and administrators, and those with other classifications not related to patient care (Table 1).

**Table 1.** Categorization of Healthcare Workers by Patient Contact Using Occupation Group Among Respondents From Boende, Democratic Republic of the Congo

Patient Contact^a^	Occupation Group	English Entries From Survey	French Entries From Survey
Direct	Nurse	Nurse, supervisor	Nurse (titulaire or supervisor), infirmier, chef de service, anesthesiste
	Physician	Physician	Physician
	Educational HCW	Student, teacher	Infirmiere en perfectionnement
	Midwife	Midwife	Sage femme, matronne
	Red cross	Paramedic, burial team	Croix rouge, secouriste
	Traditional healer	Traditional healer	Tradipraticien(ne), guerrisseur, visionnaire des esprits
	Pastor	Pastor	Pasteur, prier pour les malades
Indirect	Pharmacy	Pharmacist	Pharmacien(ne), préposée a la pharmacie
	Room attendant	Room attendant	Fille de salle, garçon de salle, intendant poste de sante
	Laboratory technician	Laboratory technician	Laborantin, technicien(ne) de labo, microscopiste
	Hygiene/housekeeping	Hygienic service	Hygiène et assainissement, désinfecter, assainissement
	Communication	Communication	Relais communautaire, assistant humanitaire
	Surveillance	Epidemiologist	Agent de surveillance, surveillance de la maladie, recherche active des cas, veterinaire
Unlikely	Guard	Sentinel	Sentinelle, garde, gardien, observateur
	Driver	Driver	Conducteur, chauffeur
	Maintenance	Maintenance	Mecanicien
	HCW (admin)	Administration	Administrateur, admnistratif, réceptionniste, secrétaire, caissier
	Ordinary worker	…	Travailleur ordinaire, TO

Abbreviation: HCW, healthcare worker.

^a^Assumed level of patient contact derived from occupation group.

### Serological Testing

#### Enzyme-Linked Immunosorbent Assays

Human anti-EBOV GP IgG and anti-EBOV NP IgG titers were measured using commercially available enzyme-linked immunosorbent assay (ELISA) kits (Alpha Diagnostic International) following the manufacturer’s protocol. The methodology has been described elsewhere [31]. Based on the manufacturer’s calibration, a sample was classified as mildly reactive if the serum antibody concentration was >1.0 units/mL control calibrator, and reactive if the titer was >2.5 units/mL. For analysis, we only include those who were considered reactive (>2.5 units/mL).

#### VP40 Reactivity via Luciferase Immunoprecipitation System

The C-terminal domain of EBOV VP40 (base pairs 583–981) was cloned into the pRen2 plasmid and transfected into Cos-1 cells generating Renilla luciferase antigen fusion proteins. Cell lysates were harvested and used in immunoprecipitation assays with protein A/G–conjugated agarose beads and test serum diluted 1:100 as described by Burbelo et al [32]. The testing procedure has been described elsewhere [31]. VP40 reactivity was determined if the relative luciferase signal postimmunoprecipitation was at least 3 standard deviations greater than the background signal, as determined from a mean of 8 previously identified VP40-seronegative samples [31].

#### Neutralization Assay

EBOV GP–bearing human immunodeficiency virus pseudotype viruses were generated as described previously [33], and pseudotype virus neutralization assays were performed [31, 34, 35]. Infection rates in presence of human serum samples were expressed as percentage of infection in presence of negative control serum. To be considered neutralizing, serum from the patients had to at least neutralize approximately 50% of virus at a dilution of 1:50 compared to appropriate control.

### Data Analysis

Results from the multiple assays were compared to explore the overlap between EBOV seroreactivity. We ran cross-tabulations on all sociodemographic factors and reported occupation to explore descriptive characteristics of the population and compared these factors to their seroprevalence for each assay. We assessed differences in factors using χ^2^ or Fisher exact test. Sensitivity analyses were conducted by including mildly reactive antibody titers with the reactive antibody titers. All analyses were completed using R 3.4.2 and SAS version 9.4 (SAS Institute) software.

### Ethical Approval

Ethical approval was obtained from the institutional review boards at the University of California, Los Angeles Fielding School of Public Health and the Kinshasa School of Public Health, DRC.

## RESULTS

### Participant Characteristics

Among the 565 HCWs, 370 (65.5%) were male and 195 (34.5%) were female (Table 2). The median age of HCWs was 40 years (interquartile range, 31.8–50 years). More than half (363 [64.2%]) of participants worked in a health facility, including the general hospital, health centers, and health posts, and 61 (10.8%) reported working in other facilities, whereas 141 (25.0%) of the participants did not identify working in any facility; this includes traditional healers, pastors, and community workers. Approximately half (279 [50.1%]) had direct contact with patients, whereas 177 (31.8%) had indirect contact and 101 (18.1%) were unlikely to have patient contact.

**Table 2.** Demographic Information and Antibody Profile for 565 Healthcare Workers in Boende, Democratic Republic of the Congo

Characteristic	Total	GP1-649 Reactive	NP Reactive	VP40 Reactive	Neutralization
Sex					
Female	195 (34.5)	31 (15.9)	17 (8.7)	14 (7.2)	3 (1.5)
Male	370 (65.5)	73 (19.7)^a^	45 (12.2)	40 (10.8)	13 (3.5)
Age, y					
<25	46 (8.2)	11 (23.9)	7 (15.2)	6 (13.0)	4 (8.7)
25–34	142 (25.4)	29 (20.4)	9 (6.3)	11 (7.7)	2 (1.4)
35–44	166 (29.6)	30 (18.1)	20 (12.0)	17 (10.2)	4 (2.4)
45–54	115 (20.5)	18 (15.7)	16 (13.9)	11 (9.6)	4 (3.4)
≥55	91 (16.2)	16 (17.6)	9 (9.9)	9 (9.9)	2 (2.2)
Facility type					
Central office	28 (5.0)	4 (14.3)	3 (10.7)	0 (0.0)	0 (0.0)
Ebola treatment center	8 (1.4)	0 (0.0)	0 (0.0)	1 (12.5)	0 (0.0)
Health center	186 (32.9)	28 (15.1)	22 (11.8)	15 (8.1)	5 (2.7)
Health post	85 (15.0)	24 (28.2)	15 (17.6)^a^	8 (9.4)	2 (2.4)
Hospital	92 (16.3)	11 (12.0)	7 (7.6)	6 (6.5)	3 (3.3)
None	141 (25.0)	32 (22.7)	13 (9.2)	19 (13.5)	6 (4.3)
Other	25 (4.4)	5 (20)	2 (8.0)^a^	5 (20.0)^a^	0 (0.0)
Patient contact					
Direct	279 (50.1)	57 (20.4)	30 (10.8)	29 (10.4)	9 (3.2)
Indirect	177 (31.8)	29 (16.4)	21 (11.9)	12 (6.8)	4 (2.3)
Unlikely	101 (18.1)	18 (17.8)^a^	10 (9.9)	12 (11.9)	3 (3.0)

Data are presented as No. (%). GP and NP reactivity was determined as >2.5 units/mL.

Abbreviations: GP, glycoprotein; NP, nucleoprotein; VP40, Ebola virus matrix protein.

^a^ *P* < .05.

### Ebolavirus Seroprevalence

**Figure 2.**
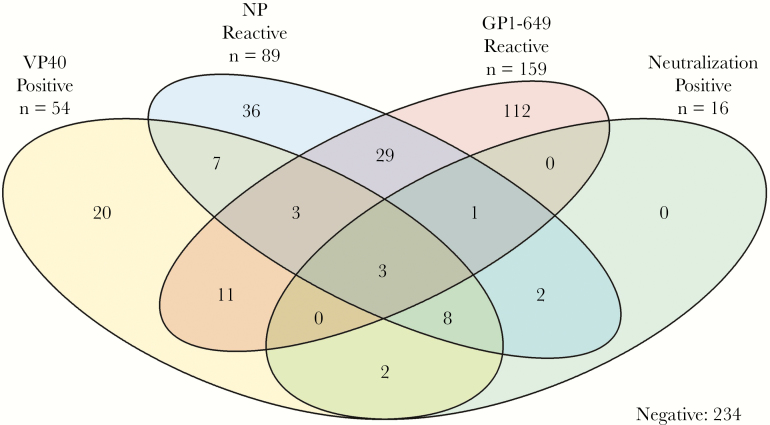
Venn diagram of overlapping immunoreactivity assay measures in healthcare workers who participated in the Boende outbreak, Democratic Republic of the Congo, 2014. Abbreviations: GP, glycoprotein; NP, nucleoprotein; VP40, Ebola virus matrix protein.

Overall, 159 (28.1%) of HCWs were GP reactive, 89 (15.8%) were NP reactive, 54 (9.5%) were VP40 reactive, and 16 (2.8%) demonstrated neutralization. Males had a higher seroprevalence compared with females regardless of the test (46.2% vs 33.3%, respectively; *P* = .01) and showed a significant difference for GP reactivity (19.7% vs 15.9%, respectively; *P* < .001) and NP reactivity (12.2% vs 8.7%, respectively; *P* = .02). While age was not statistically significant, seroreactivity for all assays decreased with increasing age. Among all HCWs, 234 (41.4%) were seroreactive for at least 1 assay (Figure 2). The greatest overlap of responses was across HCWs who were both NP and GP seropositive (n = 29 [5.1%]). Importantly, all neutralization-positive individuals were also reactive for at least 1 other distinct viral protein (ie, either VP40 and/or NP). Of those who demonstrated neutralizing capacity (n = 16), 8 presented both NP and VP40 reactivity (50.0%). However, only 3 HCWs were seroreactive for all 4 assays.

**Figure 3.**
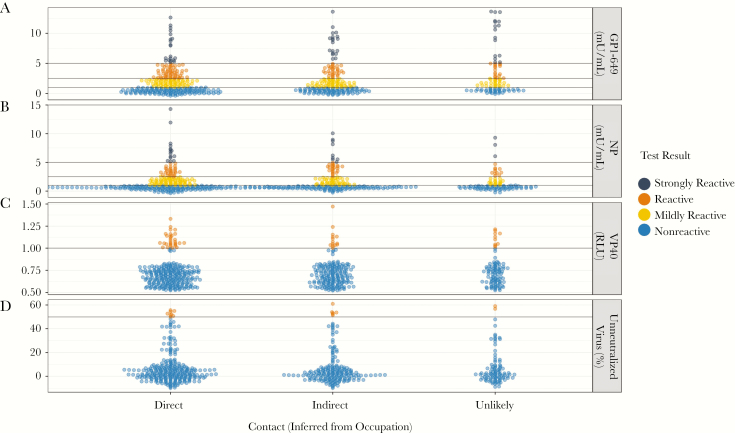
Immunoreactivity assay measures by contact type (inferred from occupation) for 565 healthcare workers. *A*, Antibody titer values measuring reactivity to Ebola virus (EBOV) glycoprotein using enzyme-linked immunosorbent assay (ELISA). *B*, Antibody titers measuring reactivity to EBOV nucleoprotein using ELISA. *C*, Relative luciferase signal obtained after immunoprecipitating EBOV VP40-Luc using participant serum samples. *D*, Neutralization capacity to at least 50% of input control. Abbreviations: GP, glycoprotein; NP, nucleoprotein; RLU, Relative Luminescence Units; VP40, Ebola virus matrix protein.

While no statistically significant differences for serologic response were observed across direct, indirect, and unlikely patient contact occupational categories, there was considerable heterogeneity in serologic response across occupation types (Table 3 and Figure 3). The scatterplot presented in Figure 3 presents the distribution of test results by HCW contact type. Among HCWs considered in direct contact with patients, traditional healers, pastors, and midwives had the highest GP and NP seroprevalence (excluding physicians due to sample size, n = 2), whereas Red Cross workers had the highest VP40 seroprevalence and neutralizing capacity (22.2% and 5.6% respectively; *P* < .05). HCWs having indirect contact with patient demonstrated similar heterogeneity across occupations. Those classified as having unlikely contacts with patients had the highest GP seroprevalence among all groups: health administrators, guards, and other (24.0%, 28.6%, and 8.0%, respectively).

**Table 3.** Antibody Profile by Contact Level (Inferred by Occupation) for Healthcare Workers in Boende, Democratic Republic of the Congo

Occupation	Total	GP1-649 Reactive	NP Reactive	VP40 Reactive	Neutralization
Direct					
Doctor	2 (0.7)	1 (50.0)	1 (50.0)	0 (0.0)	0 (0.0)
Health educator	12 (4.4)	2 (16.7)	0 (0.0)	2 (16.7)	0 (0.0)
Midwife	42 (15.3)	11 (26.2)	6 (14.3)	5 (11.9)	1 (2.4)
Nurse	155 (56.4)	24 (15.5)	14 (9.0)	13 (8.4)	4 (2.6)
Pastor	24 (8.7)	6 (25.0)	3 (12.5)	3 (12.5)	2 (8.3)
Red Cross worker	18 (6.5)	4 (22.2)	1 (5.6)	4 (22.2)	1 (5.6)
Traditional healer	22 (8.0)	8 (36.4)	3 (13.6)	3 (13.6)	0 (0.0)
Indirect					
Communication	6 (3.1)	1 (16.7)	0 (0.0)	0 (0.0)	0 (0.0)
Hygiene	76 (39.2)	12 (15.8)	12 (15.8)	8 (10.5)	3 (3.9)
Laboratory technician	5 (2.6)	2 (40.0)	0 (0.0)	0 (0.0)	0 (0.0)
Pharmacy	10 (5.2)	1 (10.0)	1 (10.0)	0 (0.0)	0 (0.0)
Room attendant	90 (46.4)	15 (16.7)	10 (11.1)	6 (6.7)	3 (3.3)
Surveillance	7 (3.6)	0 (0.0)	0 (0.0)	0 (0.0)	0 (0.0)
Unlikely					
Driver	5 (5.2)	0 (0.0)	1 (20.0)	1 (20.0)	0 (0.0)
Guard	21 (21.9)	6 (28.6)	2 (9.5)	2 (9.5)	0 (0.0)
Health administration	25 (26.0)	6 (24.0)	1 (4.0)	0 (0.0)	0 (0.0)
Maintenance	9 (9.4)	0 (0.0)	0 (0.0)	1 (11.1)	0 (0.0)
Ordinary worker	11 (11.5)	3 (27.3)	2 (18.2)	0 (0.0)	0 (0.0)
Other	25 (26.0)	2 (8.0)^a^	5 (20.0)	6 (24.0)	2 (8.0)

Data are presented as No. (%). GP and NP reactivity was determined as >2.5 units/mL.

Abbreviations: GP, glycoprotein; NP, nucleoprotein; VP40, Ebola virus matrix protein.

^a^ *P* < .05.

## DISCUSSION

Using a multiassay approach, our data suggest that at least some serological indication of previous ebolavirus exposure and/or infection among Boende HCWs is higher than previously reported [28, 35–40]. A serological survey conducted during Kikwit EBOV outbreak found prevalence of 2.2% among health workers and 9.3% in surrounding villages [41]. A recent study in Watsa in the northeastern region of the DRC reported an EBOV seroprevalence of 18.7% in the local Efe population [42].

When exploring trends by patient contact, the highest GP seroprevalence was observed in the “unlikely” contact group. However, within each category there was significant heterogeneity. These differences may be explained by the fact that, in an outbreak situation, everyone, including guards and administrators, could be required to assist with potentially infected persons when regular hospital staff are not able to meet the demands of the patients, potentially leading to exposures to bodily fluids, sick patients, or community members. This group also likely reflects the general population and may indicate that there is endemic EBOV exposure in this area. However, overall these results most likely suggest that the GP ELISA as a standalone test is relatively nonspecific. This may be due to unrelated nonspecific binding, with the high degree of glycosylation in EBOV GP lending itself to such nonspecific recognition. Alternately, it may be that GP is more sensitive than other viral proteins to the presence of cross-reactive antibodies directed against related viruses. In contrast, the unique mechanism of ebolavirus entry lends itself to highly specific, but possibly insensitive, detection of prior ebolavirus infection [43].

HCWs enrolled in this study demonstrated seroreactivity to multiple EBOV proteins. Furthermore, we identified that 16 (2.8%) of HCWs who never reported infection were not only seroreactive for at least 1 serologic test but may also be able to neutralize ebolavirus if exposed to EVD. This includes 3 individuals who were seroreactive on all tests and exhibited neutralizing capacity: a 52-year-old midwife, a 48-year-old administrator, and a 78-year-old volunteer. It is not known whether seroreactivity to assays is related to EBOV exposure during the 2014 outbreak—in particular because all HCWs included for this analysis participated in the outbreak response and reported varying levels of contact with EVD patients and biological specimens. It may also be possible that variation in antigenic response and neutralization levels may be related to asymptomatic or minimally symptomatic infection, which would not have been necessarily detected as illness due to EBOV [44]. It is possible that some seroreactive individuals could also have been exposed to a non-EBOV filovirus.

There were several limitations to this study. We were unable to interview every HCW from every health facility in the health zone, notably missing HCWs in Lokolia, which is the village in which the outbreak was concentrated, due to logistical constraints such as impassable roads. We attempted to make our study population as diverse as possible by enrolling participants in both formal and informal settings, and those who may have been displaced from their normal facility to support the outbreak response. There may have been some misclassification of specific HCW occupations, and patient contact was categorized based on occupation type and not recall of contact exposures. In addition, HCWs may play multiple different roles depending on the need of the facility during the outbreak response. We attempted to classify them based on their primary position in the health facility. Furthermore, all results are based on serologic assays, so adjusting the cutoff value can affect the overall results. Thus, we explored the range of responses (Figure 3) from each assay and used a higher manufacturer cutoff based on the calibration curve provided, assuming a higher background rate of cross-reactivity.

Our study highlights the elevated risk of HCWs in DRC and exposure to blood-borne pathogens in general. Providing adequate training of HCWs to infection control procedures and availability of personal protective equipment to reduce exposure to bodily fluids of patients they treat before an EVD outbreak occurs is ultimately one of the most important strategies to limit the spread of ebolavirus and other blood-borne pathogens of both high and low consequence. Furthermore, our findings raise additional questions about EBOV exposure, risk, and circulating virus in DRC. Currently, there is no serological gold standard for ebolavirus serology. Our results highlight the need for additional research to better understand the roles each protein plays in immune response. Better understanding of the significance of EVD seroreactivity and risk factors associated with these exposures will improve our ability to design and implement locally sustainable strategies to limit exposure of HCWs to ebolavirus and other pathogens.

## Notes

***Author contributions.*** A. W. R., N. A. H., R. D. H., B. K., P. M., and M. S. B. designed the study, supervised all aspects of study implementation, collected the data, and directed data analysis. V. H. A., R. W., D. S., C. S., A. G., and B. P. N. assisted with data cleaning and editing and also conducted data analysis and developed the figures and tables. P. M., B. I. K., E. O. W., B. P. N., J. W., M. M., and J. J. M. participated in study design and implementation. M. S. B., K. L., I. M., G. S., P. M., and B. P. N. conducted the laboratory assays. N. A. H., P. M., M. S. B., R. H. D., and A. W. R. led the manuscript writing and editing efforts.

***Acknowledgments.*** Many individuals contributed to the design of this manuscript and data collection in the field. We thank the HCWs of the Tshuapa province for their participation. We also thank Dr J. Daniel Kelly for his comments and edits on the manuscript, and Dr Alexis Mwanza, Dr Ado Bwaka, Jose Ngamboli, Nathalie Kavira, Rachel Mutombe, and the DRC Ministry of Health for their assistance with field logistics and the implementation of this study. We also thank Dr Tito Serafini and Dr Guy Cavet of Atreca, Inc, for their support of this research.

***Financial support.*** This work was supported by the Bill & Melinda Gates Foundation (award numbers OPP1066684, OPP1151786, and 20153705 [via subcontract from Atreca, Inc’s parent grant OPP1066534]); the Faucett Catalyst Fund; the National Institute of Allergy and Infectious Diseases (grant number R21AI107420 to G. S.); and a German Research Foundation (DFG) fellowship (STE 2237/1-1 to G. S.). M. S. B. is supported by the Fogarty International Center of the NIH (award number D43TW009343) and the UCGHI.

***Potential conflicts of interest.*** All authors: No reported conflicts of interest. All authors have submitted the ICMJE Form for Disclosure of Potential Conflicts of Interest. Conflicts that the editors consider relevant to the content of the manuscript have been disclosed.

Presented in part: Eighth International Symposium on Filoviruses, Antwerp, Belgium, 13 September 2016; and 65th Annual Meeting of the American Society of Tropical Medicine and Hygiene, Atlanta, Georgia, 17 November 2016.
